# U-Net Model with Transfer Learning Model as a Backbone for Segmentation of Gastrointestinal Tract

**DOI:** 10.3390/bioengineering10010119

**Published:** 2023-01-14

**Authors:** Neha Sharma, Sheifali Gupta, Deepika Koundal, Sultan Alyami, Hani Alshahrani, Yousef Asiri, Asadullah Shaikh

**Affiliations:** 1 Chitkara University Institute of Engineering and Technology, Chitkara University, Punjab 140401, India; 2 School of Computer Science, University of Petroleum & Energy Studies, Dehradun 248007, India; 3 Department Computer Science, College of Computer Science and Information Systems, Najran University, Najran 61441, Saudi Arabia; 4 Department Information Systems, College of Computer Science and Information Systems, Najran University, Najran 61441, Saudi Arabia

**Keywords:** U-Net, pretrained models, deep learning, segmentation, GI tract, radiation therapy

## Abstract

The human gastrointestinal (GI) tract is an important part of the body. According to World Health Organization (WHO) research, GI tract infections kill 1.8 million people each year. In the year 2019, almost 5 million individuals were detected with gastrointestinal disease. Radiation therapy has the potential to improve cure rates in GI cancer patients. Radiation oncologists direct X-ray beams at the tumour while avoiding the stomach and intestines. The current objective is to direct the X-ray beam toward the malignancy while avoiding the stomach and intestines in order to improve dose delivery to the tumour. This study offered a technique for segmenting GI tract organs (small bowel, big intestine, and stomach) to assist radio oncologists to treat cancer patients more quickly and accurately. The suggested model is a U-Net model designed from scratch and used for the segmentation of a small size of images to extract the local features more efficiently. Furthermore, in the proposed model, six transfer learning models were employed as the backbone of the U-Net topology. The six transfer learning models used are Inception V3, SeResNet50, VGG19, DenseNet121, InceptionResNetV2, and EfficientNet B0. The suggested model was analysed with model loss, dice coefficient, and IoU. The results specify that the suggested model outperforms all transfer learning models, with performance parameter values as 0.122 model loss, 0.8854 dice coefficient, and 0.8819 IoU.

## 1. Introduction

The human Gastrointestinal (GI) tract is an essential component of the body. The digestive system performs three main tasks: food digestion, nutrient absorption (carbohydrates, proteins, lipids, minerals, and vitamins), and waste elimination [1]. Many different illnesses can infect the GI tract. A World Health Organization (WHO) study indicates that 1.8 million fatalities annually are caused by GI tract illnesses [1]. One of the most prevalent and dangerous malignancies in the world, particularly among older males, is gastric cancer. According to GLOBOCAN 2018 data, stomach cancer is the third most dangerous malignancy and the fifth most prevalent neoplasm, accounting for an estimated 783,000 deaths in 2018. Globally, an estimated 5 million persons received a diagnosis of gastrointestinal cancer in 2019 [2]. Radiation treatment (RT) has the potential to increase the cure rates for 3.5 million patients and to relieve the symptoms of another 3.5 million patients [3]. Radiation oncologists provide X-ray beams that are aimed at the tumour while avoiding the stomach and intestines. Oncologists may observe the location of the tumour and monitor for exact dosage based on tumour cell presence, which might fluctuate from day to day, using MR-Linacs [4]. The present task is to physically delineate the location of the stomach and intestines in order to make modifications to the direction of the X-ray beam in order to enhance dosage distribution to the tumour while avoiding the other organs. Unless deep learning technologies can be used to aid automating the segmentation process, this is a laborious and time taking task that may easily extend cures from a few minutes to an hour every day.

Deep learning can assist in reducing manual work and allowing more patients to receive appropriate treatment by automating the segmentation process. Deep neural network-based methods have recently been employed for the automated diagnosis of medical illnesses [5,6]. Deep learning progress has been encouraging in recent years, with an automatic diagnosis of disorders in numerous human organs such as the brain [7], cervical cancer [8], eye problems [9], and skin cancer [10]. Deep learning is a new field of machine learning that has recently acquired interest. It has outperformed traditional algorithms in terms of accuracy since the characteristics are learned from data using a general-purpose learning technique rather than being built by human engineers [11]. The current AI boom can be attributed to the advent of deep learning. In computer vision and machine translation applications, deep networks have shown to be a significant improvement. In recent times, deep neural networks have attracted a lot of interest for use in picture segmentation. Medical imaging is only one area where deep learning-based segmentation has been put to use, along with many others, to achieve better performance. To facilitate computer-aided diagnoses and other medical analyses, the segmentation of medical pictures is a common machine vision job [12]. In this research, we investigate the GI Tract segmentation challenge. The following are the significant contributions of this research work:

- The proposed U-Net model has been deployed with six pretrained transfer learning models as a backbone to analyse its performance. The six transfer learning models chosen for the backbone of U-Net are Inception V3, SeResNet50, VGG19, DenseNet121, InceptionResNetV2, and EfficientNet B0.
- This work proposed a U-Net model based on deep learning that has been created for the small size of images so that local features for segmentation can be enhanced and extracted efficiently.
- The proposed U-Net model has been deployed on the UW-Madison GI tract image segmentation dataset for the stomach, small bowel, and large bowel segmentation in the GI tract.
- Model performance metrics such as model loss, dice coefficient, and IoU coefficient are used to evaluate the models.

The remaining manuscript is arranged as follows: Section 2 presents a brief review of earlier work in this area, Section 3 defines the proposed methodology, Section 4 represents the results and discussion, and Section 5 concludes the paper and discusses the major findings.

## 2. Related Work

Over the past few years, highly encouraging outcomes have been produced in the medical imaging field employing computer-aided-diagnosis algorithms [13,14,15,16]. A review of the works revealed that several programmed algorithms based on both handmade approaches and deep learning methods have been extensively utilised to identify and categorize GI tract abnormalities [17,18,19,20,21]. Naqvi et al. [17] utilised the KVASIR dataset to assess their work on a system to identify GI illnesses. The authors employed six visual characteristics to construct the smoothness of the picture, which was created using Haralick features and Local Binary Patterns (LBP). Following feature collection, they train the model via kernel discriminant study and Logistic Regression. They received an F1 score of 0.75 [18], extracted picture characteristics using Bidirectional Marginal Fisher Analysis (BMFA), and supplied them to a Support Vector Machine (SVM) for the purpose of classification. Further, transfer learning was applied with data augmentation to the KVASIR dataset [19]. The pre-trained network Inception V3 was used to fine-tune the dataset. The model was 91.5% accurate. Zhang et al. [20] developed a CNN-based technique for the categorization of stomach precancerous anomalies such as ulcers, erosion, and polyp. They employed an iterative reinforcement learning method with SqueezeNet to reduce the computing time and size of the model. The total accuracy was 88.90%. In Ref. [21], Inception V3 and VGGNet pre-trained models on ImageNet dataset were utilised in the features extraction phase, and SVM was employed for classification; the mixture of the mined features produces extreme moral outcomes. Pogorelov et al. [22] tested 17 alternative techniques before settling on a mixture of the transfer learning model ResNet50 and the Logistic Model Tree (LMT) classifier. The primary goal of this work is to optimise the performance of our CNN model while minimising computing interval and assets for the job of categorising 8 classes as illness states, therapeutic processes, or structural innovations [22].

Gibson, E. published a registration-free neural network model for segmenting right organs in 2018 [23]. This includes the pancreas, the digestive system (oesophagus, stomach, and duodenum), and the adjacent organs, which are essential for routing in endoscopic biliary and pancreatic processes (liver, spleen, left kidney, and gallbladder). In 2020, Wang et al. introduced a multi-scale deep network (MCNet) for complete gastrointestinal (GI) lesion segmentation from endoscopic photos. To help train models, this network takes in information from both the global and local levels [24]. In 2020, Khan et al. proposed a deep learning-based technique to classify or identify ulcers, polyps, and bleeding in the gastrointestinal tract. In Ref. [25], it was suggested that an altered Recurrent Convolutional Neural Network (RCNN) be used for ulcer segmentation. Using the direct extension, Galdran et al. 2021 developed a method for semantic segmentation of standard encoder–decoder networks applicable to delineating gastrointestinal polyps from endoscopic images. In addition, 600 annotated frames of gastrointestinal (GI) operation equipment were released in 2021 as part of a proposal by Jha, D. et al. [26] to raise the bar and spur more research. The goal of this research is to utilize existing knowledge of the problem of GI tract segmentation. There has been limited research on the segmentation of GI tract organs. This work intends to propose a U-Net model designed from scratch for segmenting organs such as the stomach, large bowel, and small bowel. Furthermore, the proposed model has been compared with different models utilising various transfer learning models as backbones of U-Net topologies.

## 3. Proposed Methodology

**Figure 1:**
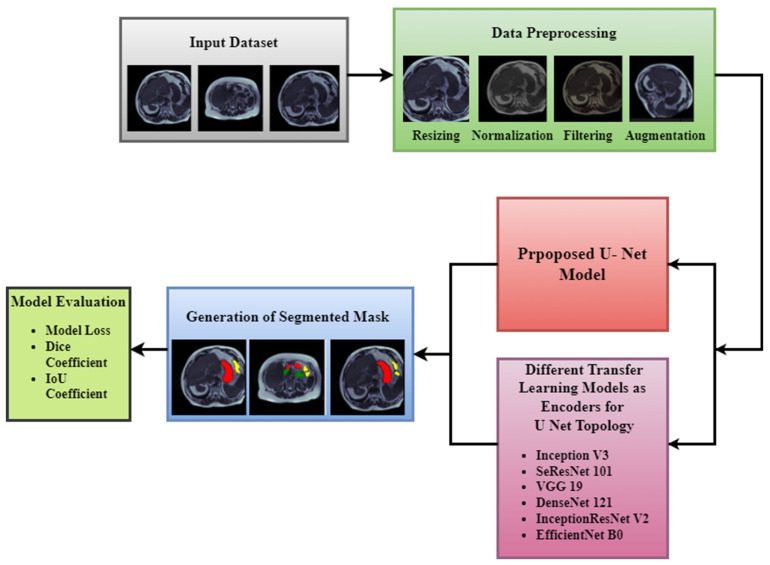
Proposed Methodology for GI Tract Segmentation.

The main objective of this work is to propose a U-Net model designed from scratch for accurate segmentation of healthy organs to assist the radio-oncologist. This section describes the proposed methodology for the segmentation of the GI tract. Section 3.1 will present the input dataset used for the task of segmentation. Section 3.2 discusses various pre-processing steps applied in order to enhance the dataset for further processing. Section 3.3 shows the suggested U-Net model which is designed from scratch. Section 3.4 discusses the various transfer learning models which are used as the backbones of the U-Net model. Figure 1 shows our proposed methodology for GI tract segmentation.

### 3.1. Input Dataset

**Figure 2:**
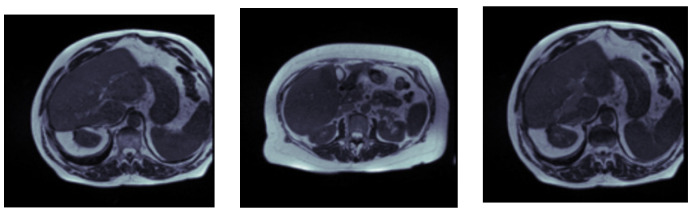
Sample Images of the Dataset.

The public land-grant research university Institution of Wisconsin-Madison in Madison, Wisconsin, has released an MRI scan dataset. In the dataset, 85 people had scans for 1 to 6 days. For different patients, every scan has either 144 or 80 slices. There are 38,496 photos in the collection as a whole. Figure 2 shows a few examples of the images in the set. The RLE encoding format is used for the images in the dataset. With the help of deep learning algorithms, these RLE-encoded images are used to make masks for small bowel, large bowel, and stomach.

### 3.2. Dataset Pre-Processing

Dataset pre-processing is performed to enhance the data. It is used to make data more compatible for further stages. The pre-processing steps performed in this research are resizing, filtering, normalization, and augmentation. A detailed description of various pre-processing steps is shown in the further sections.

#### 3.2.1. Resizing

**Figure 3:**
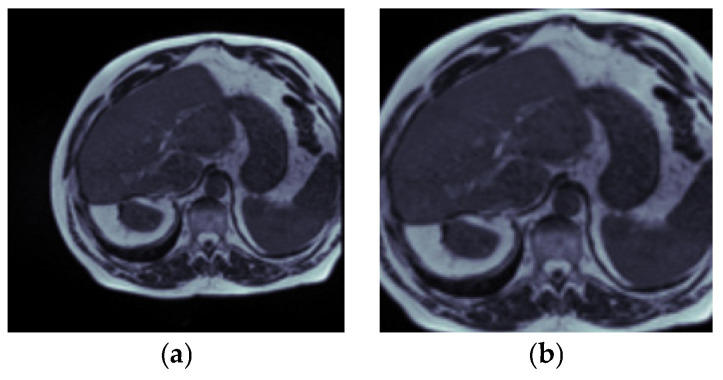
Dataset Resizing: (**a**) Input Image & (**b**) Resized Image.

Resizing is the process of converting all the images in the dataset to a predefined size. Since the neural network receives images of the same size, we have to resize the dataset. The size of the image after applying resizing step is 160 × 160 × 1. Figure 3a shows an input image and Figure 3b shows its resized image.

#### 3.2.2. Gaussian Filter

**Figure 4:**
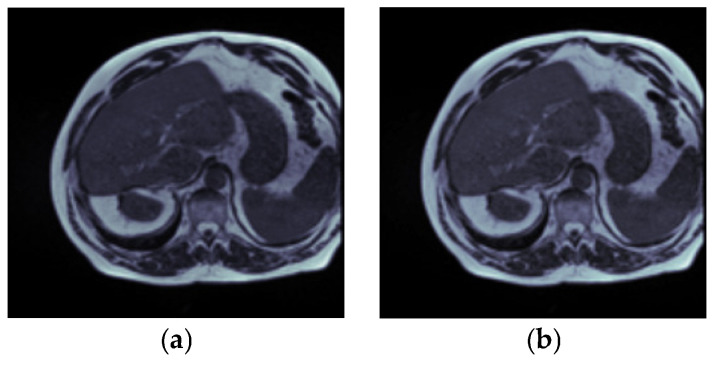
Filtering: (**a**) Input Image & (**b**) Filtered Image.

A low pass filter called a Gaussian is used to blur certain portions of an image and lessen noise (high-frequency components). As implied by the name of the function, the weights inside the kernel, which are used to calculate the weighted average of the nearest points (pixels) in a picture, have the form of a Gaussian distribution. Figure 4a displays an input image that was chosen at random, and Figure 4b displays the filtered image.

#### 3.2.3. Normalization

**Figure 5:**
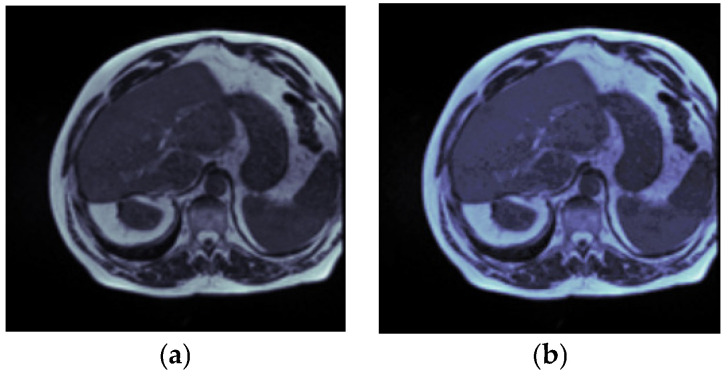
Data Normalization: (**a**) Input Image, (**b**) Normalized image.

Normalization is the process of converting all the pixel values of the picture in the range of 0 to 1. It is done by dividing each pixel value by 255. The Figure 5a shows the input image and Figure 5b shows normalized image.

#### 3.2.4. Augmentation

Data augmentation is a pre-processing method that is applied to increase the diversity of data and to increase the number of images in the dataset only for visual perception during training. It is also called a dataset regularizer because it makes the dataset more diverse. It is used to increase the images by applying modifications while keeping the class label intact.

**Figure 6:**
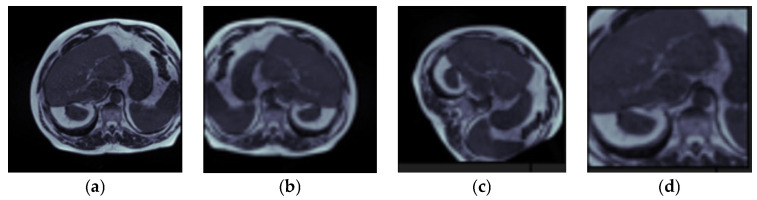
Data Augmentation: (**a**) Input Image, (**b**) Horizontal Flip, (**c**) Rotation & (**d**) Zoom.

The augmentations techniques used in the dataset are: horizontal flip, rotation by 120 degrees, and zoom by 0.2. Figure 6 shows the original and augmented images from the dataset: Figure 6a original images, Figure 6b horizontal flip, Figure 6c rotation, and Figure 6d zoom.

### 3.3. Segmentation Using Proposed U-Net Model

For the segmentation of biomedical images, Olaf Ronneberger et al. [27] created the U-Net. There are two ways to the architecture. The first path, also known as the contraction path or encoder, is used to extract the context from the picture. A standard stack of convolutional and max pooling layers serves as the encoder. In order to achieve exact localization using transposed convolutions, the second path—also known as the decoder—is the symmetric expanding path. U-Net is its moniker because of how it resembles the letter U. As a result, it is an end-to-end fully convolutional network (FCN), which means that it only comprises convolutional layers and lacks any dense layers, allowing it to accept images of any size.

**Figure 7:**
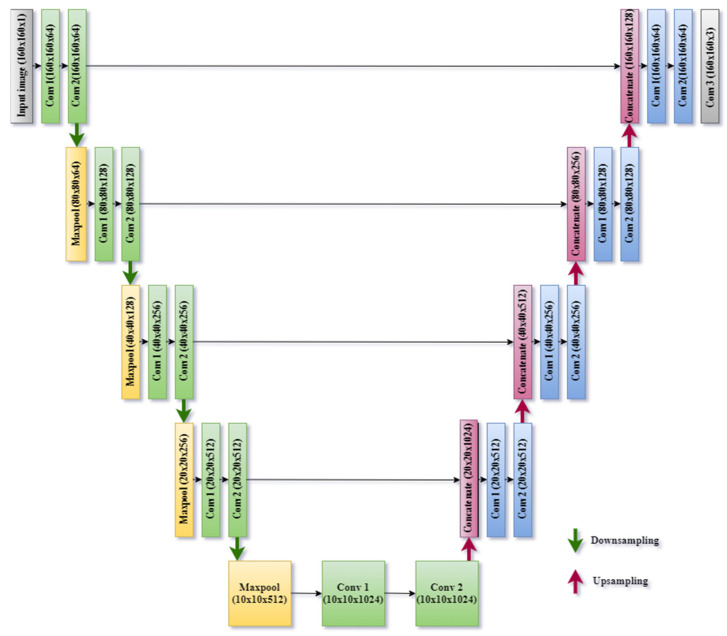
U-Net Model from Scratch.

In this research work, a U-Net Model has been used for the automatic segmentation of the stomach, large bowel, and small bowel in the gastrointestinal tract. The layer of the proposed U-Net model has been designed from scratch. The number of layers is selected from the experimentation. A lot of experimentation has been done for selecting the number and sequence of layers in the U-Net model and the best combination of layers that gives the highest results has been selected for the segmentation of the GI tract. The proposed U-Net model contains a combination of convolution and max pool layers. Figure 7 shows the block diagram of the different layer used in the suggested U-Net model. As discussed earlier, the U-Net has two branches, one encoder and one decoder; the proposed model also contains two branches: the left one is an encoder and the right one is the decoder. It forms the shape of the English alphabet U; that is why it is called U-Net. There is no dense layer in the model: it is only a combination of convolution, max pool, and transpose convolution layers. The U-Net model proposed in this work has entirely different image sizes as compared with the standard U-Net model. The input image size in the proposed study is 160 × 160. Different layers of this U-Net model have been designed according to the input image size.

Table 1 shows the model summary of the layers used in the designed U-Net model. The input size of the images for the model is 160 × 160 × 1. This input image will pass through four downsampling blocks. Each down-sampling block contains a combination of two convolutions and one max pool layer. The input size, output size, number of filters, size of the filter, and number of parameters for each layer have been represented in Table 1. The size of the filter is 3 × 3 for each convolution layer and 2 × 2 for each max pool layer. The number of filters is increasing or doubles in each convolution block as 64, 128, 256, and 512. A centre block is also used in the model which is the same as the downsampling block. It has 1024 filters for 2 convolution layers. Four downsampling and one centre block collectively make the encoder branch. The size of the image after passing through the encoder branch becomes 10 × 10 × 1024.

The decoder branch of this model contains transposed convolution, concatenation, and convolution layers. As the image size is reduced in the encoder branch, the decoder branch is used to increase the size of the image. It contains four upsampling blocks; the size of the image will increase and the number of filters will be reduced after every up-sampling block. The number of filters in the decoder branch is reduced as 1024, 512, 256, 128, and 64. A final convolution layer is also used, which makes the size of the image 160 × 160 × 3. The input image to the encoder branch is 10 × 10 × 1024, and the output size of the image after going through all the upsampling blocks is 160 × 160 × 3. The size of the output image is identical to the input image, but at the output of the U-Net model, we get the segmented image. The segmented image contains a mask having three objects: small bowel, large bowel, and stomach.

### 3.4. Segmentation Using Pre-Trained Transfer Learning Models

In general, transfer learning refers to a process where a model developed for one problem is used in some capacity for another related problem. Transfer learning is a deep learning method where a neural network structure is first trained on a dataset that is identical to the one that is being cracked. A new model that is trained on the target issue then incorporates one or more layers from the trained model. In this work, the segmentation of the GI tract was performed using six pre-trained transfer learning models: Inception V3 [28], SeResNet50 [29], VGG19 [30], DenseNet121 [31], InceptionResNetV2 [32], and Efficient Net B0 [33]. The U-Net topology was built using these pre-trained models. The inception V3 [24] model is only an improved and efficient form of the inception V1 architecture. The Inception V3 model uses a number of strategies to optimise the network for improved model adaption. It is more productive. In comparison to the Inception V1 and V2 models, it has a deeper network, but its speed is unaffected. It costs less to compute. It uses auxiliary Classifiers as regularizers. SE ResNet [29] is a ResNet version that uses squeeze-and-excitation blocks to allow the system to the bringactivechannel–wisestatistic arrangement. Squeeze-and-Excitation Networks (SENets) provide a CNN building block that enhances channel interdependencies at essentially no computational cost. They participated in the ImageNet competition and contributed to a 25% increase in performance over the previous year. In addition to providing a significant speed improvement, they are simple to include in current designs. One of the simplest CNN designs utilised in ImageNet contests is VGG19 (Visual Geometry Group-19) [30]. The architecture comprises 16 convolutional layers, 3 fully connected layers, and a total of 19 layers to learn weights and bias parameters; thus, the name VGG-19. With the exception of three more convolution layers, the VGG-19 design is remarkably identical to the VGG-16 architecture. The most recent VGG model is VGG19. By leveraging smaller links among the layers, the DenseNet (Dense Convolutional Network) design [31] aims to make deep learning networks even deeper while also improving their training efficiency. Each layer of the convolutional neural network is linked to all layers below it; thus, the first layer is connected to layers 2 through 4 and so on, and layers 2 through 5 and so on. To maximise information flow across the network’s tiers, this is done. Each layer receives input from all the earlier levels and transmits its individual feature maps to all the layers that will follow it in order to maintain the feed-forward nature. In addition to the fundamental convolutional and pooling layers, the model is composed of two significant components. These are the Transition layers and the Dense Blocks. Inception-ResNet combines the Inception architecture with residual connections. A convolutional neural network called Inception-ResNet-v2 was trained using more than a million images from the ImageNet dataset. The network has 164 layers and can categorise photos into 1000 different item types. It is constructed using a mix of the Residual connection and the Inception structure. Multiple convolutional filters of different sizes are merged with residual networks in the Inception–Resnet block. The introduction of residual links not only escapes the humiliation problem brought on by deep arrangements but also shortens training time. Using a compound coefficient, the convolutional neural network design and scaling technique known as EfficientNet B0 consistently scales all measurements of depth, breadth, and resolution. The EfficientNet scaling method consistently increases network breadth, depth, and resolution using a set of present scaling constants, in contrast to standard practice, which scales these elements arbitrarily. In the accumulation of squeeze-and-excitation blocks, the foundational EfficientNet-B0 system is built on the MobileNetV2 inverted bottleneck residual blocks. Table 2 compares the six transfer learning models in terms of the number of layers, parameters, and processing time.

## 4. Results and Discussion

The proposed model (U-Net from scratch) and six pre-trained models, such as Inception V3, SeResNet50, VGG19, DenseNet121, InceptionResNetV2, and EfficientNet B0, were examined in this work, and the Keras Tensorflow Package was used to generate the models. Keras is a freely available, simple-to-use framework designed exclusively for NNs. It is open source and works with both Theano and Tensorflow. It is purposely built to accelerate DNN computations. All simulations in this study were conducted on the Google Colab Platform, utilizing a Colabnotebook quipped with Tensorflow and a GPU.

### 4.1. Hyperparameter Tuning

With a batch size of 32, the models were trained for 20 epochs. The batch size hyper-parameter specifies the number of samples to proceed before updating the model’s internal parameters, whereas the epochs parameter specifies the number of runs over the whole training data. The critical hyper-parameter is the learning rate, which controls the model’s learning speed. It should not be very high or excessively low. If the learning rate is set too low, the network may take an inordinate time to attain the minimum loss, or if it is set too high, the network may overshoot the low-loss regions. The learning rate has been set to 0.0001 in this work. For model compilation, the Adam [34] optimization method was utilized. Additionally, all convolutional layers have been activated using the ReLU [35] activation function.

### 4.2. Analysis of Training and Validation Loss

**Figure 8:**
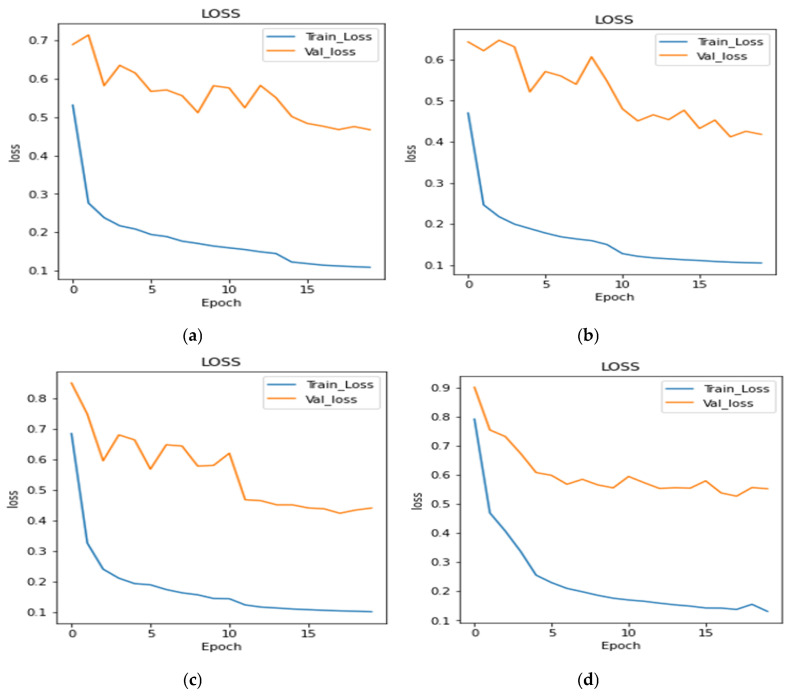

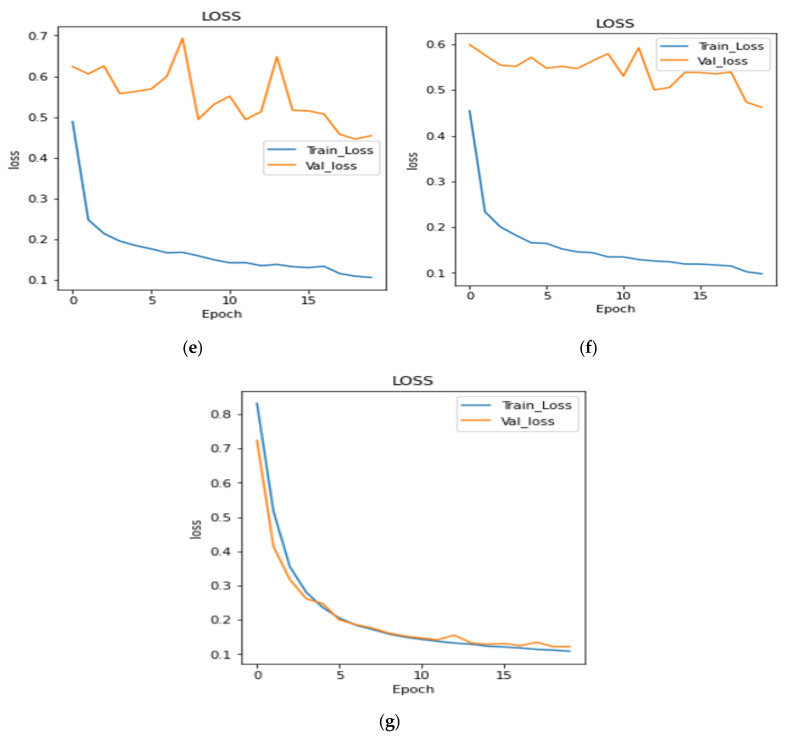
Loss Plots: (**a**) DenseNet121, (**b**) Inception V3, (**c**) SeResNet50, (**d**) VGG19, (**e**) InceptionResNetV2, (**f**) EfficientNet B2, & (**g**) Proposed U-Net.

The proposed model and transfer learning models were evaluated in terms of model loss, dice coefficient and IoU coefficient. Figure 8a to Figure 8g shows the loss curves during training and validation for all the transfer learning and proposed models, respectively. From the figure, it can be concluded that the transfer learning models perform similarly. The plots of transfer learning models are very much similar, but the plot shown in Figure 8g is very uniform. The proposed model achieved less loss as compared to transfer learning models.

**Figure 9:**
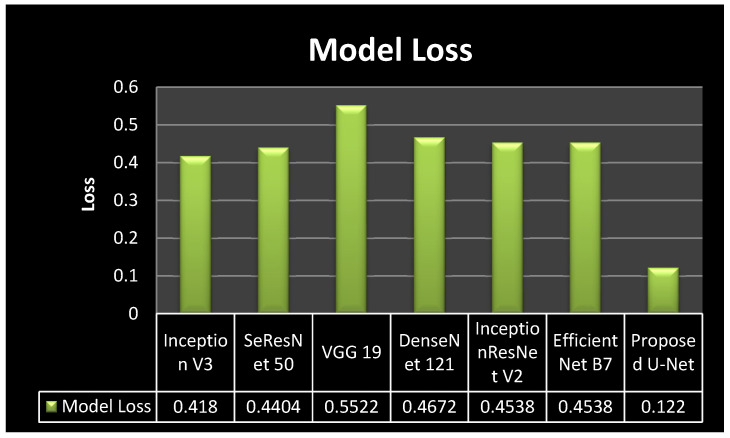
Model Loss Comparison Graph.

Figure 9 shows the comparison of model loss in a graphical form. It can be seen from Figure 9 that all six transfer learning models achieved similar results. The transfer learning models achieved the model loss values as 0.418 for inception v3, 0.4404 for SeResNet 50, 0.5522 for VGG 19, 0.4672 for DenseNet121, 0.4538 for Inception ResNetV2, and 0.4538 for Efficient Net B0. The proposed model achieved a 0.122 model loss value. It can be concluded from the figure that the proposed model outperforms all the transfer learning models, as it achieved the least loss value.

### 4.3. Analysis of Dice Coefficient

**Figure 10:**
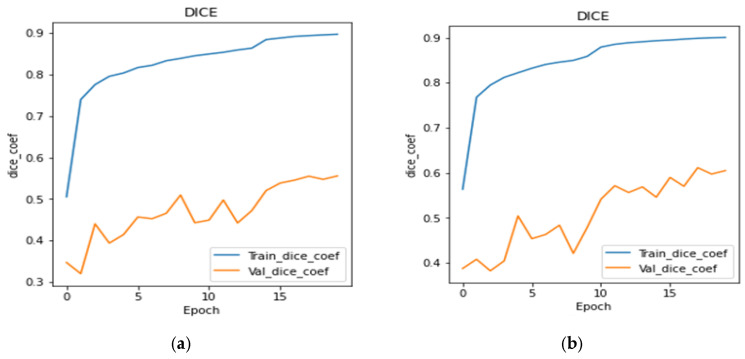

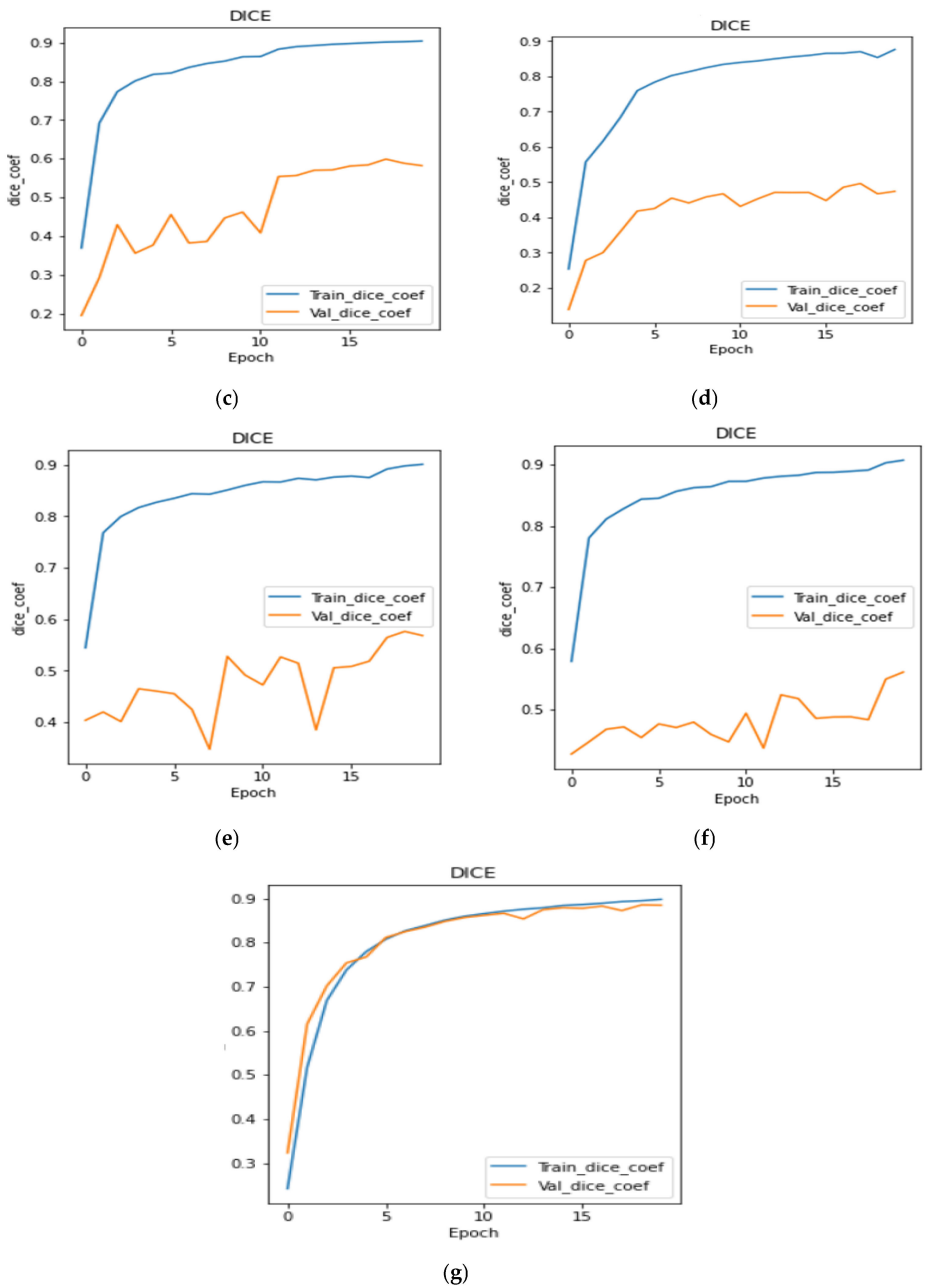
Dice Coefficient Plots: (**a**) DenseNet121, (**b**) Inception V3, (**c**) SeResNet50, (**d**) VGG19, (**e**) InceptionResNetV2, (**f**) EfficientNet B2, & (**g**) Proposed U-Net.

Figure 10a–g shows the dice coefficient curves during training as well as validation for all the transfer learning and proposed models, respectively. From the figure, it can be concluded that all six transfer learning models show almost similar performance. The dice coefficient plots of transfer learning models are very much similar, but the plot shown in Figure 10g gives the best performance among all the models. The proposed model achieved the highest dice coefficient as compared to transfer learning models.

**Figure 11:**
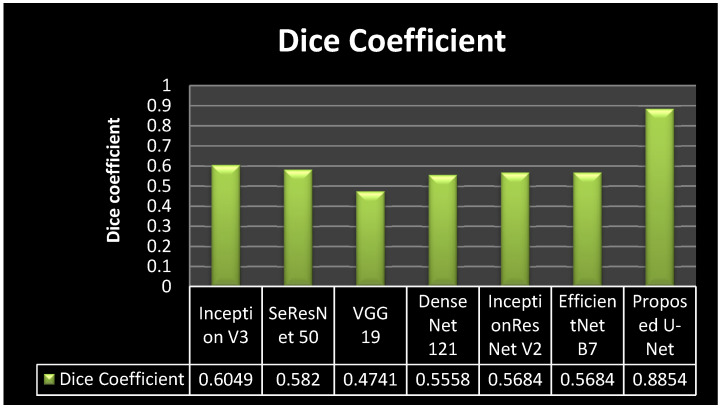
Dice Coefficient Comparison Graph.

Figure 11 shows the comparison of model loss in a graphical form. It can be seen from Figure 11 that all six transfer learning models achieved similar results. The transfer learning models achieved the model loss values as 0.6049 for inception v3, 0.582 for SeResNet 50, 0.4741 for VGG 19, 0.5558 for DenseNet121, 0.5684 for Inception ResNetV2, and 0.5684 for Efficient Net B0. The proposed model achieved a 0.8854 model loss value. It can be concluded from the figure that the proposed model outperforms all the transfer learning models, as it achieved the highest dice coefficient value.

### 4.4. Analysis of IoU Coefficient

**Figure 12:**
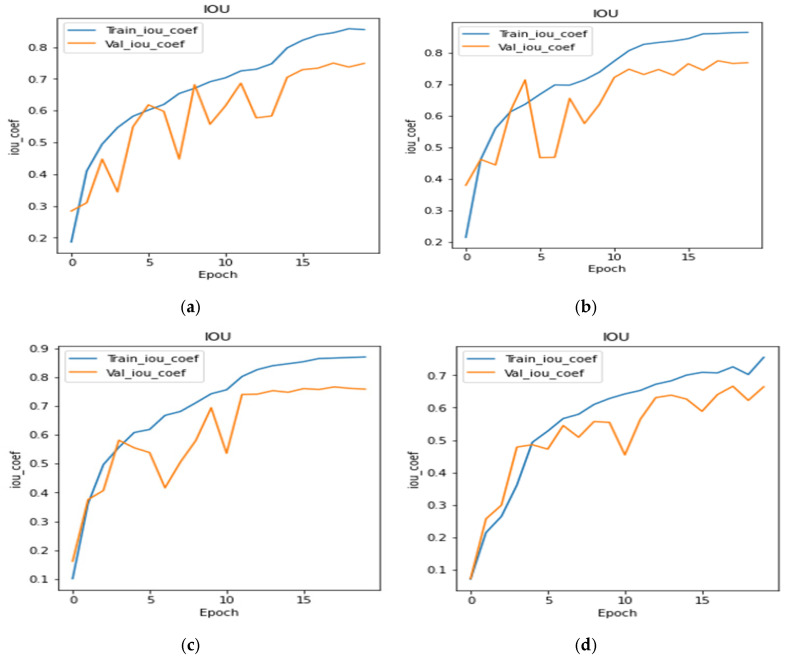

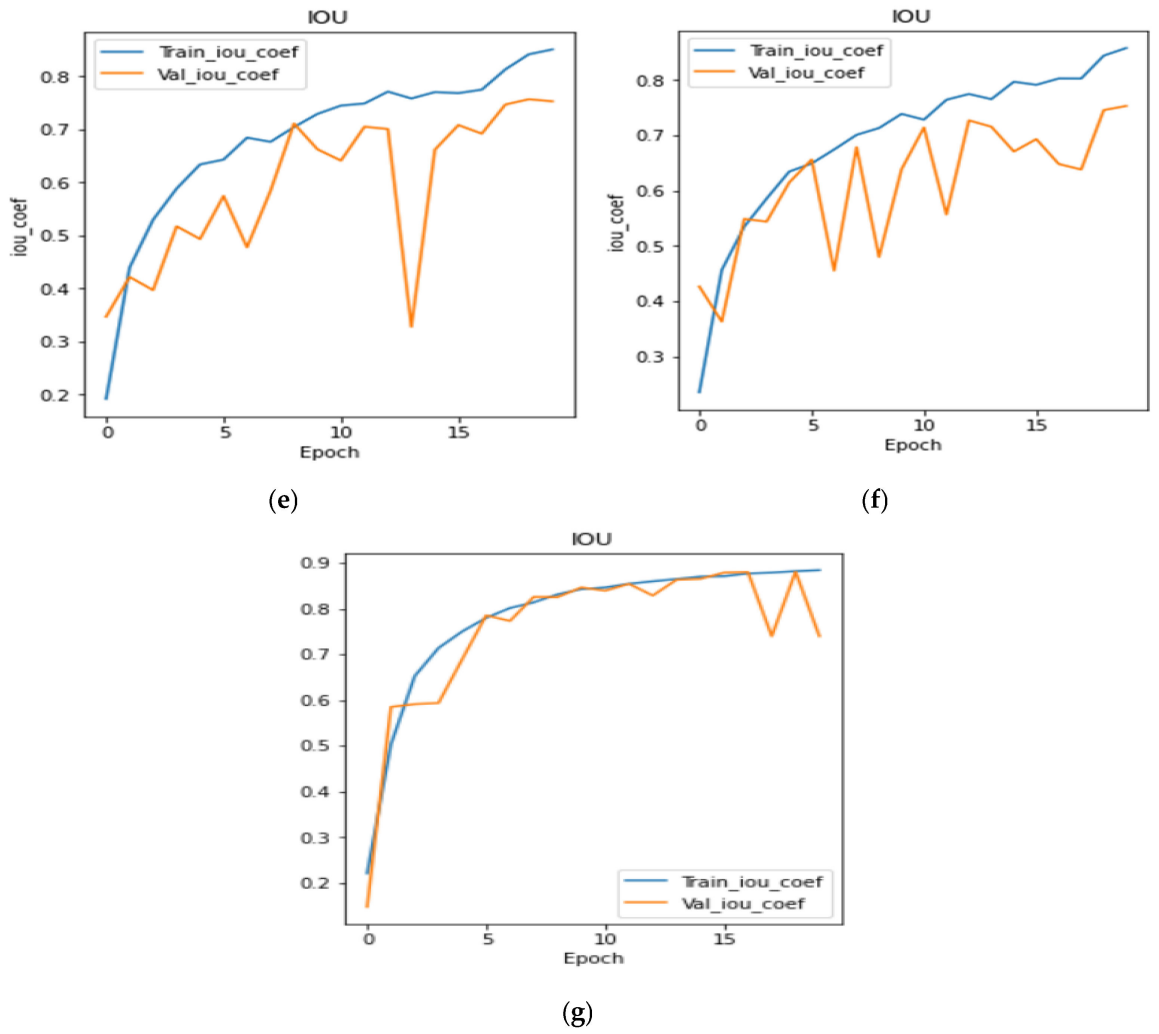
IoU Coefficient Plots: (**a**) DenseNet121, (**b**) Inception V3, (**c**) SeResNet50, (**d**) VGG19, (**e**) InceptionResNetV2, (**f**) EfficientNet B2, & (**g**) Proposed U-Net.

Figure 12a to Figure 12g shows the IoU curves during training and validation for all the transfer learning and proposed models, respectively. From the figure, it can be concluded that the transfer learning models perform similarly. The plots of transfer learning models are very much similar, but the plot shown in Figure 12g is very much uniform. The proposed model achieved the highest IoU as compared to transfer learning models.

**Figure 13:**
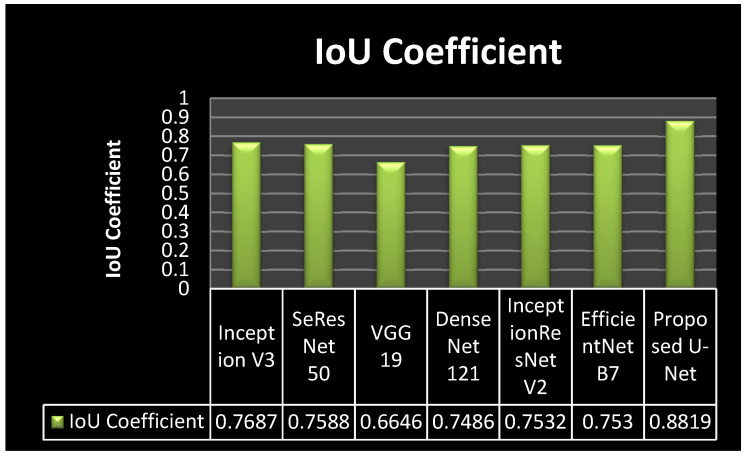
IoU Coefficient Comparison Graph.

Figure 13 shows the comparison of the IoU coefficient in a graphical form. It can be seen from Figure 13 that all six transfer learning models achieved similar results. The transfer learning models achieved the IoU values as 0.7687 for inception v3, 0.7588 for SeResNet 50, 0.6646 for VGG 19, 0.7486 for DenseNet121, 0.7532 for Inception ResNetV2, and 0.753 for Efficient Net B0. The proposed model achieved a 0.8819 model loss value. It can be concluded from the figure that the proposed model outperforms all the transfer learning models as it achieved the highest IoU value.

### 4.5. Visual Analysis of Segmented Images

**Figure 14:**
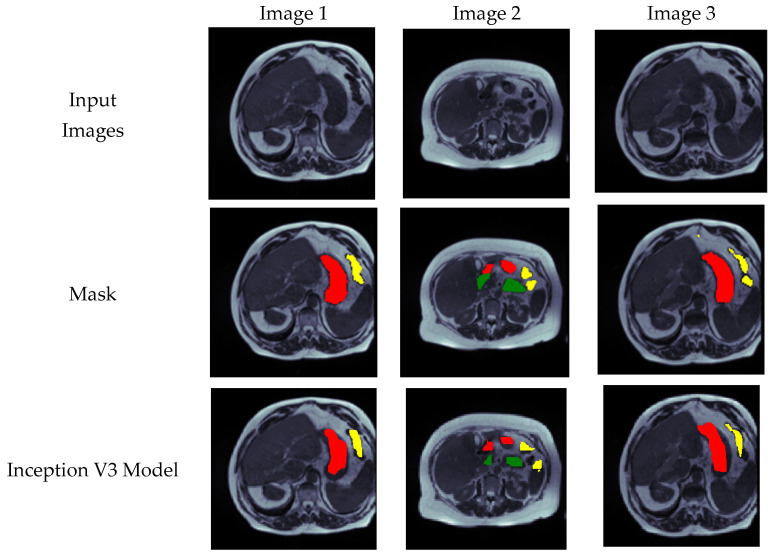

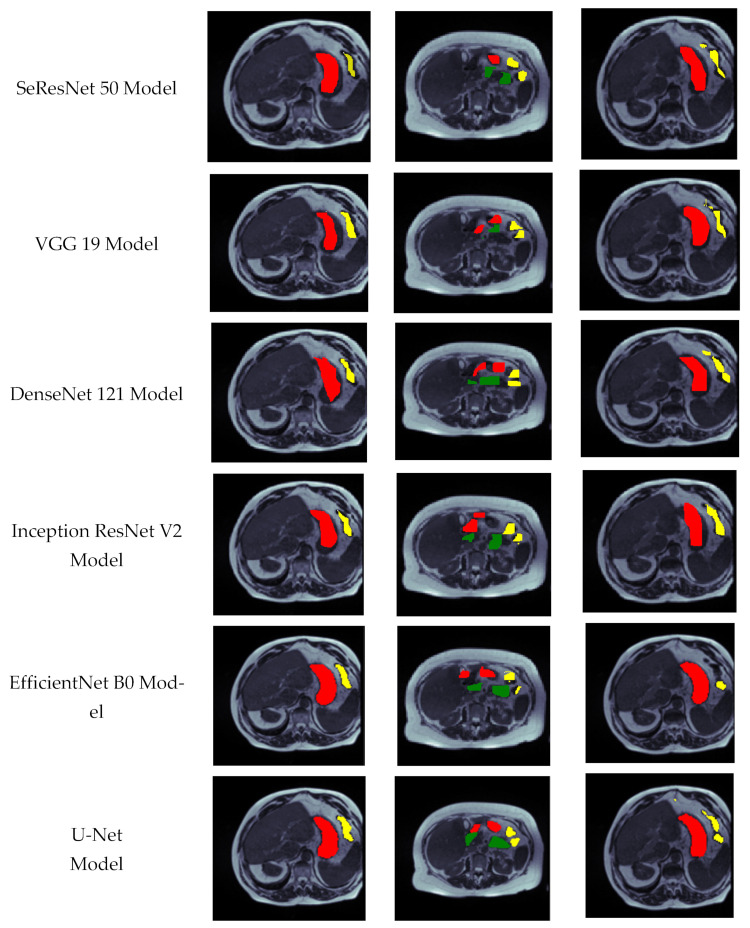
Visual analysis of the results.

Figure 14 shows results in the form of images. The figure includes the input images and the mask for the respective images which are obtained using RLE encoding. The figure also includes the predicted images by using all the transfer learning models and the proposed model. Here red color shows the stomach, green shows large bowel and yellow color shows small bowel. From the figure, it can be seen that the images predicted by the proposed models are very much similar to the original mask.

We have analysed the results in terms of model loss, dice coefficient, and IoU. The results were also visualized in the form of images and their respective masks obtained by the proposed and transfer learning models. From all the results discussed previously, it can be concluded that the proposed model shows the best results as compared to all the six transfer learning models. The proposed model achieved the results of 0.122 model loss, 0.8854 dice coefficient, and 0.8819 IoU.

## 5. Conclusions

Gastrointestinal cancer cases are increasing every year according to the reports of the WHO and GLOBOCAN. GI cancer can be treated with the help of radiation treatment. In radiation treatment, the radiation oncologist must manually outline the position of the tumour while avoiding other healthy organs like the stomach and intestine so that high-power X-ray beams can be directed toward the tumour. This paper proposed a deep learning-based technique that can help radiation oncologists to automatically segment the stomach and intestine. The paper proposed a U-Net model designed from scratch and this model has been compared with six pretrained transfer learning models, namely, Inception V3, SeResNet50, VGG19, DenseNet121, InceptionResNetV2, and EfficientNet B0, which were used as the backbone for the U-Net topology. The comparison shows that the proposed U-Net model whose layers are designed to form scratch outperforms all the transfer learning models.

## Figures and Tables

**Table 1:** Model Summary of the Proposed U-Net Model.

Name of the Block	Name of the Layer	Input Image Size	Filter Size	Number of Filters	Activation Function	Output Image Size	Number of Parameters
Downsampling Block 1	Input image	160 × 160 × 1	-----	-----	-----	160 × 160 × 1	-----
	Conv1	160 × 160 × 1	3 × 3	64	ReLU	160 × 160 × 64	640
	Conv2	160 × 160 × 64	3 × 3	64	ReLU	160 × 160 × 64	36,928
Downsampling Block 2	Maxpool	160 × 160 × 64	2 × 2	64	-----	80 × 80 × 64	-----
	Conv1	80 × 80 × 64	3 × 3	128	ReLU	80 × 80 × 128	73,856
	Conv2	80 × 80 × 128	3 × 3	128	ReLU	80 × 80 × 128	147,584
Downsampling Block 3	Maxpool	80 × 80 × 128	2 × 2	128	-----	40 × 40 × 128	-----
	Conv1	40 × 40 × 128	3 × 3	256	ReLU	40 × 40 × 256	295,168
	Conv2	40 × 40 × 256	3 × 3	256	ReLU	40 × 40 × 256	590,080
Downsampling Block 4	Maxpool	40 × 40 × 256	2 × 2	256	-----	20 × 20 × 256	-----
	Conv1	20 × 20 × 256	3 × 3	512	ReLU	20 × 20 × 512	1,180,160
	Conv2	20 × 20 × 512	3 × 3	512	ReLU	20 × 20 × 512	2,359,808
Center Block	Maxpool	20 × 20 × 512	2 × 2	512	-----	10 × 10 × 512	-----
	Conv1	10 × 10 × 512	3 × 3	1024	ReLU	10 × 10 × 1024	4,719,616
	Conv2	10 × 10 × 1024	3 × 3	1024	ReLU	10 × 10 × 1024	9,438,208
Upsampling Block 1	Concatenate	10 × 10 × 1024	-----	1-----	-----	20 × 20 × 1024	-----
	Conv1	20 × 20 × 1024	3 × 3	512	ReLU	20 × 20 × 512	9,438,208
	Conv2	20 × 20 × 512	3 × 3	512	ReLU	20 × 20 × 512	2,359,808
Upsampling Block 2	Concatenate	20 × 20 × 512	-----	-----	-----	40 × 40 × 512	-----
	Conv1	40 × 40 × 512	3 × 3	512	ReLU	40 × 40 × 512	2,359,808
	Conv2	40 × 40 × 512	3 × 3	512	ReLU	40 × 40 × 512	590,080
Upsampling Block 3	Concatenate	40 × 40 × 512	-----	-----	-----	80 × 80 × 256	-----
	Conv1	80 × 80 × 256	3 × 3	128	ReLU	80 × 80 × 128	590,080
	Conv2	80 × 80 × 128	3 × 3	128	ReLU	80 × 80 × 128	147,584
Upsampling Block 4	Concatenate	80 × 80 × 128	-----	-----	-----	160 × 160 × 128	-----
	Conv1	160 × 160 × 128	3 × 3	64	ReLU	160 × 160 × 64	147,584
	Conv2	160 × 160 × 64	3 × 3	64	ReLU	160 × 160 × 64	36,928
	Conv3	160 × 160 × 64	3 × 3	3	ReLU	160 × 160 × 3	195

Total Parameters: 34,512,323, Trainable Parameters: 34,512,323, Non-trainable Parameters: 0.

**Table 2:** Comparison of Transfer Learning Models.

Model Name	Number of Layers	Number of Parameters (Million)	Processing Time (Hours)
Inception V3 [28]	48	25	4:36
SeResNet50 [29]	101	44.5	5:18
VGG 19 [30]	19	138	5:35
DenseNet 121 [31]	121	29	4:46
Inception ResNet V2 [32]	164	56	5:10
EfficientNet B0 [33]	237	11	5:25

## Data Availability

Not applicable.
